# The effects of raspberry consumption on anthropometric indices and liver function tests in adults: a GRADE-assessed systematic review and meta-analysis

**DOI:** 10.3389/fnut.2024.1419417

**Published:** 2024-08-05

**Authors:** Mostafa Shahraki Jazinaki, Hanieh Barghchi, Pegah Rahbarinejad, Naseh Pahlavani

**Affiliations:** ^1^Student Research Committee, Mashhad University of Medical Sciences, Mashhad, Iran; ^2^Department of Nutrition, Faculty of Medicine, Mashhad University of Medical Sciences, Mashhad, Iran; ^3^Health Sciences Research Center, Torbat Heydariyeh University of Medical Sciences, Torbat-e Heydariyeh, Iran; ^4^Social Determinants of Health Research Center, Torbat Heydariyeh University of Medical Sciences, Torbat-e Heydariyeh, Iran

**Keywords:** raspberry, anthropometric indices, liver function tests, systematic review, meta-analysis

## Abstract

**Background:**

In recent years, the therapeutic effects of raspberry have been the subject of several randomized control trials (RCTs). However, there is no consensus about the effect of raspberry consumption on anthropometric indices and liver function tests. So, the present review aims to investigate this effectiveness by conducting a meta-analysis.

**Methods:**

PubMed, Web of Science, and Scopus databases were searched to identify eligible randomized control trials (RCTs) up to March 2024. The overall effect sizes were estimated using the random-effects model as weighted mean difference (WMD) and 95% confidence intervals (CIs).

**Results:**

Nine studies (10 arms) with 355 participants were eligible for inclusion in this review. This meta-analysis showed that raspberry consumption did not lead to significant changes in anthropometric indices (weight, body mass index, and waist circumference) and liver function tests (aspartate aminotransferase and alanine aminotransferase).

**Conclusion:**

This review revealed that raspberry consumption had no significant impact on anthropometric indices or liver function tests. However, more RCTs with a larger sample size and higher sensitivity are needed to draw definite conclusions.

**Systematic review registration**: https://www.crd.york.ac.uk/prospero/display_record.php?RecordID=477568, PROSPERO ID: CRD42023477568.

## 1 Introduction

Anthropometric indices and liver function tests are considered predictors of metabolic homeostasis dysregulation, encompassing a spectrum of abnormalities such as non-alcoholic fatty liver (NAFLD) development, type 2 diabetes (T2DM), and atherosclerotic disease (1). The 2020 International Expert Consensus Statement introduced the concept of metabolic dysfunction-associated fatty liver disease (MAFLD), highlighting that individuals with MAFLD exhibit more metabolic disorder traits than those with NAFLD (2). Additionally, obesity which is an important component of MAFLD, also has been linked to the incidence of MAFLD (1).

Diet plays a crucial role in influencing metabolic disorders, and prolonged consumption of a nutritionally poor diet with low bioactive components can predispose individuals to metabolic dysregulation (3, 4). Epidemiological studies indicate that dietary habits are challenging to change over the long term (5, 6). Notably, simple nutritional interventions should be considered, especially those aimed at increasing dietary fiber and phytochemical content with antioxidant and anti-inflammatory properties (6). In this context, there is a growing body of evidence supporting the role of berry fruits in preventing and controlling metabolic disorders (6). Besides polyphenol-containing foods and beverages, raspberry stands out for its flavonoids, tannins, anthocyanins, phenolic compounds, tyrosol, ellagitannins, and resveratrol (7). Previous research has explored the impact of raspberry consumption on various aspects such as immune-metabolic response profiles (8), vascular endothelial function (9), arterial stiffness (10), blood pressure (11), postprandial glucose, and acute and chronic inflammation (12). These studies have proved the health-promoting effect of raspberry consumption (8–12). In addition to these studies, both *in vitro* and *ex vivo* research have confirmed the beneficial effects of raspberry components and the entire fruit (13). Raspberry, with its pharmaceutically active components, demonstrates anti-inflammatory, anti-oxidative, and anti-atherosclerotic properties by inhibiting the formation of reactive oxygen species (ROS) and increasing the synthesis of inducible nitric oxide synthase (iNOS) and endothelial nitric oxide synthase (eNOS) (14). Although the effectiveness of raspberry consumption on human health has been investigated in various studies (8–12), no systematic review and meta-analysis study has examined the impact of raspberry consumption on anthropometric indices including weight, body mass index (BMI), waist circumference (WC), and liver function tests including aspartate aminotransferase (AST) and alanine aminotransferase (ALT) in adults. Therefore, the present systematic review and meta-analysis aim to investigate the effects of raspberry consumption on these parameters.

## 2 Materials and methods

### 2.1 Search strategy

All stages of designing and conducting this systematic review were done based on the PRISMA (Preferred Reporting Items for Systematic Review and Meta-analysis) framework (15). The protocol for conducting this review is registered in the PROSPERO database with the register ID CRD42023477568 (Date of registration: 11/11/2023).

The question of this review was designed based on the PICOS framework (16). Population (adults aged 18 and older), Intervention (Raspberry consumption), Comparison (control group), Outcome (changes of liver function tests or anthropometric indices), and Study Design (RCTs).

### 2.2 Study selection

PubMed, Scopus, and ISI (Web of Science) databases were comprehensively searched until March 2024 to find RCTs investigating the effect of Raspberry on anthropometric indicators and liver function tests. The search strategy implemented in databases consisting of MeSH and non-MeSH terms was as follows:

(“Raspberry” OR “*Rubus Occidentalis*” OR “*Rubus idaeus*” “Rubus coreanus”) AND (“Liver enzymes” OR “Hepatic enzymes” OR “Aspartate aminotransferase” OR “AST” OR “Alanine aminotransferase” OR “ALT” OR “Alkaline phosphatase” OR “ALP” OR “Gamma-glutamyl transpeptidase” OR “GGT” OR “Body Weight” OR “Body Mass Index” OR “BMI” OR “Weight Loss” OR “obesity” OR “Waist Circumference” OR “WC” OR “Hip Circumference” OR “HC” “Quetelet Index” OR “Weight Reduction” OR “overweight” OR “fat mass” OR “Body Fat”) AND (“randomized” OR “placebo” OR “Clinical Trial “OR “randomly” OR “trial” OR “randomized controlled trial” OR “RCT” OR “Controlled Clinical Trial”). The details of the search strategy in each database are provided in Supplementary Table 1.

This search did not include any time and language restrictions. To reduce the possibility of missing related studies, the reference list of all related studies and the Google Scholar search engine were also checked.

### 2.3 Eligibility criteria

Two researchers (H.B and M.Sh.J) independently screened the papers found from the initial search based on the inclusion criteria in order to find relevant studies.

All eligible studies met the following criteria: (a) human RCT studies on adults, (b) intervention with any form of raspberry, (c) definition of an appropriate control group, and (d) reporting changes in liver function tests or anthropometric indices during the intervention.

#### 2.3.1 Exclusion criteria

Animal research, non-RCT studies including observational studies, review articles, short communications, letters to the editor, studies without a proper control group, combination therapy, and conducting an intervention on a population less than 18 years old were the exclusion criteria of this review.

### 2.4 Data extraction

Information related to the objectives of this meta-analysis was extracted from the eligible studies by two authors (H.B and M.Sh.J) independently. The extracted items include the name of the first author, the year of publication, the country where trials were conducted, sample size and number of participants in each group, the type and dose of received raspberry and the duration of the intervention, the type of intervention in the control group, the characteristics of the participants (mean age, mean BMI and health status) and mean changes and standard deviation (SD) of liver function markers and anthropometric indices. Disputed items were discussed until a consensus was reached.

### 2.5 Risk of bias assessment

The risk of bias assessment of included studies was done using the Revised Cochrane risk of bias tool for randomized trials (ROB 2) by two authors (P.R and M.Sh.J), independently (17). This tool assesses the risk of bias in the following five main domains: Bias arising from the randomization process, Bias due to missing outcome data, Bias in selection of the reported result, Bias due to deviations from intended interventions, Bias in measurement of the outcome. The risk of bias in each domain was classified into 3 levels: low, Some concerns, and high. The general risk of bias was considered as high in cases where the high risk of bias item was 1≤ or some concerns items were 2≤. Also, if the number of some concerns item was 1, the general risk of bias determinates as some concerns, and if the risk of bias in all domains was low, the general risk of bias was considered as low. Disagreements were resolved in consultation with the third researcher (N.P).

### 2.6 Data synthesis and statistical analysis

All analyses were performed using STATA software, version 17 (Stata Corp, College Station, TX, United States), and *p*-values <0.05 were considered as the level of statistical significance.

In this meta-analysis, the overall effect sizes were presented as weighted mean differences (WMD) and 95% confidence interval (CI). The pooled effect size was estimated using the random effect model method proposed by DerSimonian and Laird (18) and employed mean changes and the standard deviation (SD) of both intervention and control groups. Using the method of Hozo et al. (19) 95% confidence intervals (CIs), standard errors (SEs), and interquartile ranges (IQRs) were converted to SDs. In cases where the mean changes were not reported directly, they were estimated by subtracting the outcome values at the beginning of the intervention from the end of the intervention (mean change = final values − baseline values). Also, SD changes were calculated using the following formula in case of non-reporting: Change SD = square root [(SDbaseline)^2^ + (SDfinal)^2^ − (2 × R × SDbaseline × SDfinal)] (20). The heterogeneity of included studies was evaluated using Cochran’s *Q* test and measured using *I*-squared statistic (*I*^2^). *I*^2^ > 50% or *p*-value <0.05 was deemed as significant heterogeneity (21). A subgroup analysis was performed to find the source of heterogeneity among the pooled studies to evaluate the effect of raspberry consumption on each outcome based on the following predefined criteria (22): Country (Korea and non-Korea), gender (Both sexes, Males, and Females), age (≤35 and >35 years), type of intervention (black raspberry and None-black raspberry), duration of the intervention (≤8 and >8 weeks), health status, baseline BMI (Normal, Overweight, and Obesity), and baseline level of some outcomes including BMI, AST (≤30, and >30 mg/dL) and ALT (≤30 and >30 mg/dL). Publication bias among eligible studies was evaluated by applying Egger’s regression test and visual interpretation of funnel plots (23). The effectiveness of the overall effect sizes from each of the included effect sizes was checked by performing a sensitivity analysis using the leave-one-out method (24, 25).

### 2.7 GRADE assessment

The quality of the reviewed evidence in this meta-analysis was evaluated based on the GRADE (Grading of Recommendations Assessment, Development, and Evaluation) protocol (26). Based on the GRADE framework, the quality of the studies was evaluated according to five types of limitations, including the Risk of bias (27), Inconsistency, Indirectness, Imprecision, and Publication bias.

## 3 Results

### 3.1 Study selection

**Figure 1:**
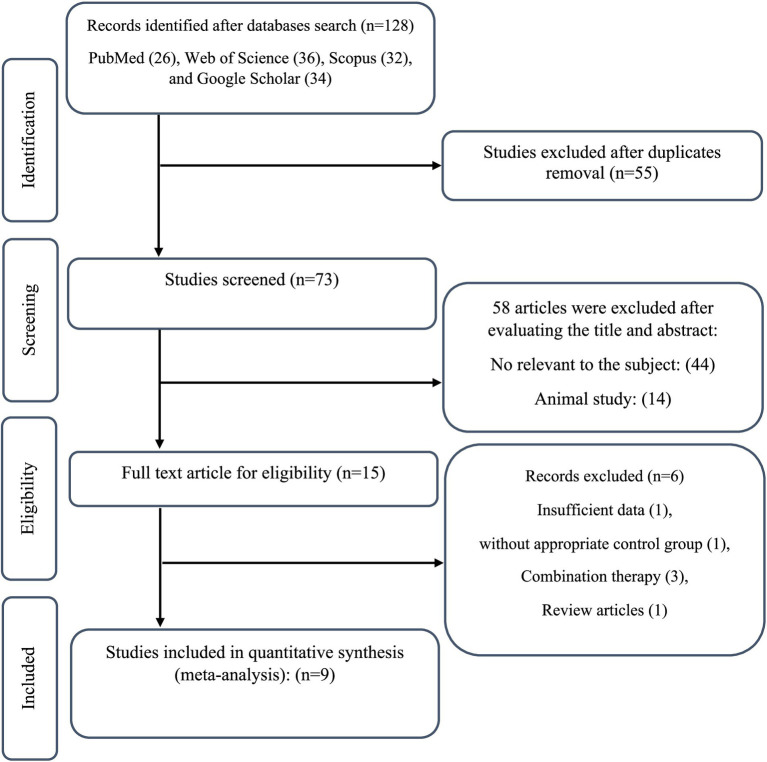
Flowchart of study selection for inclusion trials in the systematic review.

A total of 128 studies were obtained by conducting a comprehensive search. After removing 55 duplicate papers, 73 studies were screened based on their titles and abstracts as a result 58 papers were excluded. Then, 15 full-text articles were read according to the requirement, and then 6 studies were excluded due to not meeting the inclusion criteria. Finally, 9 studies (10 arms) with 355 participants were eligible for inclusion in this systematic review (Figure 1) (8, 12, 28–34).

### 3.2 Study characteristics

The included studies were published between 2015 (28–30), and 2023 (34). The design of one study was a crossover (12), while others had a parallel design. The sample size of the studies varied from 22 (12), to 77 individuals (32). Among the included studies, 2 were conducted only on females (29, 30), and 2 only on males (28, 34), while the others were conducted on both sexes. The mean age of the participants was between 24.65 (28) and 63.8 years (34), and the mean BMI varied from 23.52 (32) to 35.3 kg/m^2^ (12). The intervention populations included women with obesity (29, 30), healthy men (28) and individuals with type 2 diabetes (12), pre-diabetes (31), at risk of metabolic syndrome (8, 33), borderline-high cholesterol levels (32), and andropause symptoms (34). The duration of the intervention varied from 4 (12, 28), to 12 weeks (10, 29–32). The type of intervention in the included trials was raspberry ketones in 2 (29, 30), red raspberry in 1 (12), and black raspberry in 4 (28, 31, 32, 34). In contrast, the exact type of raspberry was not mentioned in the 2 studies (8, 33). Control groups consumed a placebo in only 4 included studies (28, 31, 32, 34), and in the other trials, the control groups received no intervention (8, 12, 29, 30, 33). The characteristics of the included studies are shown in Table 1.

**Table 1:** Characteristic of included studies in meta-analysis.

Studies	Country	Study design	Participant	Sample size and sex	Sample size		Trial duration (Week)	Means Age		Means BMI		Intervention		Reported outcomes
					IG	CG		IG	CG	IG	CG	Raspberries dose (mg/d)	Control group	
Park et al. 2015 (28)	Korea	Parallel, R, PC, DB	Healthy male smokers	39 M	20	19	4	24.5 ± 2.6	24.8 ± 2.9	20–25	20–25	Freeze-dried black raspberry (*Rubus coreanus*) 30 g/d	Placebo	Body weight, WC
Khazaal et al. 2015 (29)	Iraq	Parallel, R, PC, SB	Women with obesity	38 F	20	18	12	31.75 ± 5.58	32.72 ± 7.00	35.41 ± 3.34	34.83 ± 2.99	Raspberry ketones 0.5 g/d	Without treatment	Body weight, BMI, WC
Mosah et al. 2015 (30)	Iraq	Parallel, R, PC, SB	Women with obesity	38 F	20	18	12	31.75 ± 5.58	32.72 ± 7.00	35.41 ± 3.34	34.83 ± 2.99	Raspberry ketones 0.5 g/d	Without treatment	AST, ALT
An et al. 2016a (31)	Korea	Parallel, R, PC, DB	Subjects with Prediabetes	24 M and F	12	12	12	60.2 ± 8.6	58.4 ± 8.3	24.4 ± 2.3	24.4 ± 1.9	Low-dose black raspberry (*Rubus occidentalis*) 0.9 g/d	Placebo	BMI, WC, AST, ALT
An et al. 2016b (31)	Korea	Parallel, R, PC, DB	Subjects with Prediabetes.	27 M and F	15	12	12	58.4 ± 7.4	58.4 ± 8.3	25.0 ± 2.1	24.4 ± 1.9	High-dose black raspberry (*Rubus occidentalis*) 1.8 g/d	Placebo	BMI, WC, AST, ALT
Schell et al. 2019 (12)	USA	Cross over, R, PC, DB	Adults with Type 2 Diabetes	22 M and F	22	22	4	54 ± 19.69	54 ± 19.69	35.3 ± 9.38	35.3 ± 9.38	Frozen red raspberries 250 g/d	Maintained their usual diet	AST, ALT
Cho et al. 2020 (32)	Korea	Parallel, R, PC, DB	Individuals with Borderline-high cholesterol levels	77 M and F	39	38	12	47.03 ± 12.30	47.61 ± 12.20	23.47 ± 2.99	23.58 ± 3.26	Freeze-dried unripe black raspberry (*Rubus croreanus*) extract 0.6 g/d	Placebo	Body weight, BMI, WC
Franck et al. 2020 (8)	Canada	Parallel, R, PC, DB	Subjects at risk of metabolic syndrome	48 M and F	24	24	8	32.46 ± 10.12	31.92 ± 8.05	30.42 ± 5.00	29.38 ± 3.94	Frozen raspberries 280 g/d	Maintained their health and food habits stable	BMI, WC
Franck et al. 2022 (33)	Canada	Parallel, R, PC, DB	Participants with overweight or abdominal obesity, and with slight hyperinsulinemia or hypertriglyceridemia	24 M and F	13	11	8	32.6 ± 10.5	34.0 ± 9.5	29.3 ± 3.9	32.8 ± 5.7	Frozen raspberries 280 g/d	Maintained their usual diet	BMI, WC
Jung et al. 2023 (34)	Korea	Parallel, R, PC, DB	Men with andropause symptoms	30 M	15	15	12	66.13 ± 6.16	61.47 ± 7.65	24.9 ± 1.95	25.79 ± 1.97	Unripe Black Raspberry Extract 4.8 g/d	Placebo	Body weight, BMI, WC

IG, intervention group; CG, control group; DB, double-blinded; SB, single-blinded; PC, placebo-controlled; CO, controlled; R, randomized; NR, not reported; F, female; M, male; NR, not reported; WC, waist circumference; AST, aspartate transaminase; ALT, alanine transaminase.

### 3.3 Risk of bias assessment

**Figure 2:**
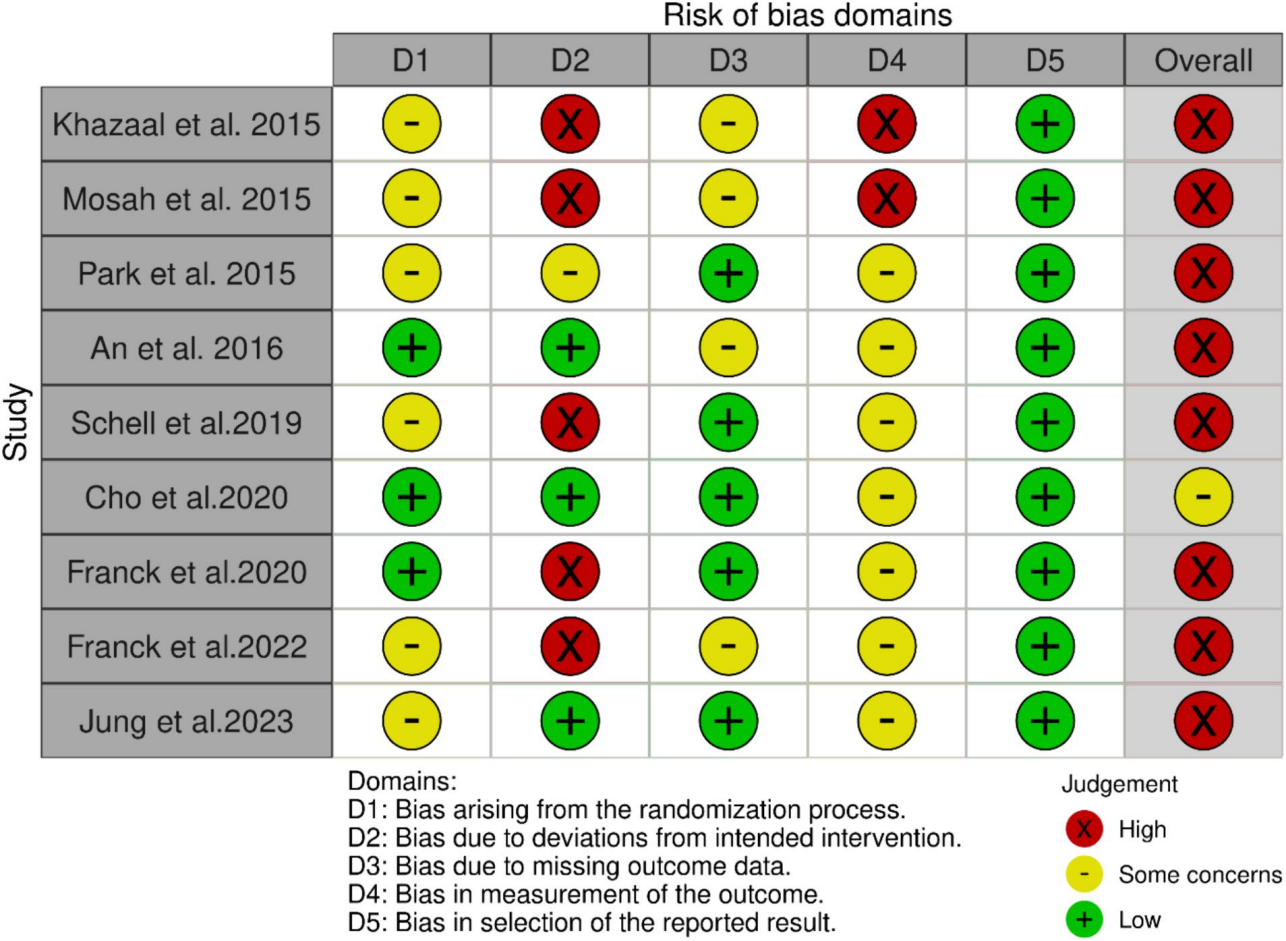
Risk of bias assessment plot.

The quality of included intervention studies was evaluated using the ROB 2 tool (17). The general risk of bias in one trial identified as some concerns (32) while the rest of the studies had a high general risk of bias. The risk of bias assessment plot in Figure 2 and the summary plot of the risk of bias assessment in Supplementary Figure 2 are shown.

### 3.4 Meta-analysis

#### 3.4.1 Effect of raspberry consumption on weight

**Figure 3:**
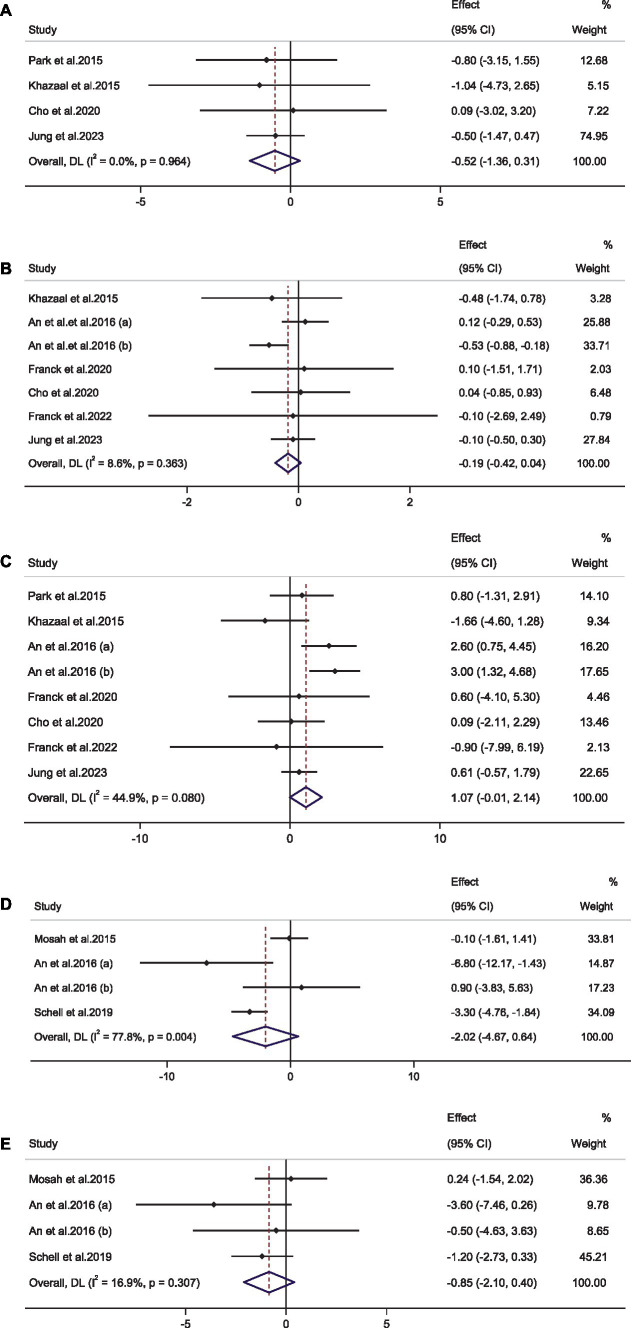
**(A–E)** Forest plot representing weighted mean difference and 95% confidence intervals (CIs) for the effect of the raspberry consumption on the **(A)** weight (Kg); **(B)** body mass index (Kg/m^2^); **(C)** waist circumference (cm); **(D)** aspartate transaminase (U/L), and **(E)** alanine transaminase (U/L).

Pooling 4 effect sizes with 184 participants showed that raspberry consumption had no significant effect on weight compared to the control groups [WMD, −0.52 Kg; 95%CI, (−1.36 to 0.31); *p* = 0.22; 184 participants] (Figure 3A). Furthermore, no significant heterogeneity was observed among the included studies (*I*^2^ = 0.0%, *p* = 0.96). A subgroup analysis was performed to find sources of heterogeneity, but it did not show a significant effect of raspberry on weight in any predefined criteria (Table 2).

**Table 2:** Subgroup analyses of raspberry consumption on anthropometric indices and liver function tests in adults.

	NO	WMD (95%CI)	*P*-value	Heterogeneity		
				*P* heterogeneity	*I*^2^	*P* between sub-groups
Subgroup analyses of raspberry consumption on weight (Kg)						
Overall effect	4	−0.52(−1.36, 0.31)	0.22	0.96	0.0%	
Country						
Korea	3	−0.49(−1.35, 0.36)	0.25	0.90	0.0%	0.77
None-Korea	1	−1.04 (−4.73, 2.65)	0.58	–	–	
Duration (weeks)						
≤8	1	−0.80(−3.15, 1.55)	0.50	–	–	0.80
>8	3	−0.48(−1.37, 0.41)	0.29	0.89	0.0%	
Raspberry type						
Black raspberry	3	−0.49(−1.35, 0.36)	0.25	0.90	0.0%	0.77
None-black raspberry	1	−1.04(−4.73, 2.65)	0.58	–	–	
Health status						
Healthy	2	−0.86(−2.85, 1.11)	0.39	0.91	0.0%	0.87
Andropause symptoms	1	−0.50(−1.46, 0.46)	0.31	–	–	
Borderline-high cholesterol	1	0.09(−3.02, 3.20)	0.95	–	–	
Age						
≤35	2	−0.86(−2.85, 1.11)	0.39	0.91	0.0%	0.70
>35	2	−0.44(−1.37, 0.47)	0.34	0.72	0.0%	
Gender						
Both sexes	2	0.09(−3.02, 3.20)	0.95	–	–	0.89
Male	1	−0.54(−1.43, 0.35)	0.23	0.81	0.0%	
Female	1	−1.04(−4.73, 2.65)	0.58	–	–	
Baseline BMI (Kg/m^2^)						
Normal	1	−0.80 (−3.15, 1.55)	0.50	–	–	0.90
Overweight	1	0.09 (−3.02, 3.20)	0.95	–	–	
Obesity	2	−0.53 (−1.47, 0.40)	0.26	0.78	0.0%	
Subgroup analyses of raspberry consumption on BMI (Kg/m^2^)						
Overall effect	7	−0.18(−0.41, 0.04)	0.11	0.36	8.6%	
Country						
Korea	4	−0.16 (−0.48, 0.16)	0.33	0.10	51.8%	0.87
None-Korea	3	−0.23 (−1.16, 0.68)	0.61	0.85	0.0%	
Duration (weeks)						
≤8	2	0.04(−1.32, 1.41)	0.94	0.89	0.0%	0.75
>8	5	−0.18(−0.47, 0.11)	0.22	0.17	37.7%	
Raspberry type						
Black raspberry	4	−0.16(−0.48, 0.16)	0.33	0.10	51.8%	0.87
None-black raspberry	3	−0.23(−1.16, 0.68)	0.61	0.85	0.0%	
Health status						
Healthy	1	−0.48(−1.73, 0.77)	0.45	–	–	
Prediabetes	2	−0.21(−0.85, 0.42)	0.50	**0.01**	81.9%	0.96
Healthy	1	−0.48(−1.73, 0.77)	0.45	–	–	
At Risk of METS	2	0.04(−1.32, 1.41)	0.94	0.89	0.0%	
Andropause symptoms	1	−0.10(−0.49, 0.29)	0.62	–	–	
Borderline-high cholesterol	1	0.04(−0.84, 0.92)	0.92	–	–	
Age						
≤35	3	−0.23(−1.16, 0.68)	0.61	0.85	0.0%	0.87
>35	4	−0.16(-0.48, 0.16)	0.33	0.10	51.8%	
Gender						
Both sexes	5	−0.16(−0.54, 0.21)	0.38	0.19	34.4%	0.84
Male	1	−0.10(−0.49, 0.29)	0.62	–	–	
Female	1	−0.48(−1.73, 0.77)	0.45	–	–	
Baseline BMI (Kg/m^2^)						
Overweight	3	−0.16 (−0.66, 0.32)	0.50	0.05	66.2%	0.88
Obesity	4	−0.12 (−0.48, 0.24)	0.51	0.94	0.0%	
Subgroup analyses of raspberry consumption on WC (cm)						
Overall effect	8	1.06(−0.005, 2.13)	0.05	0.08	44.9%	
Country						
Korea	5	1.44 (0.30, 2.58)	**0.01**	0.07	52.8%	0.06
None-Korea	3	−1.01 (−3.36, 1.33)	0.39	0.72	0.0%	
Duration						
≤8	3	0.65(−1.20, 2.51)	0.49	0.90	0.0%	0.68
>8	5	1.14(−0.30, 2.58)	0.12	0.01	67.1%	
Raspberry type						
Black raspberry	5	1.44(0.30, 2.58)	**0.01**	0.07	52.8%	0.06
None-black raspberry	3	−1.01(−3.36, 1.33)	0.39	0.72	0.0%	
Health status						
Healthy	2	−0.21(−2.58, 2.16)	0.86	0.18	43.7%	**0.03**
Prediabetes	2	2.81(1.57, 4.06)	**<0.001**	0.75	0.0%	
At risk of METS	2	0.14(−3.77, 4.05)	0.94	0.72	0.0%	
Borderline-high cholesterol	1	0.09(−2.11, 2.29)	0.93	–	–	
Andropause symptoms	1	0.61(−0.57, 1.79)	0.31	–	–	
Age						
≤35	4	−0.01(−1.58, 1.56)	0.98	0.59	0.0%	0.13
>35	4	1.57(0.19, 2.96)	**0.02**	0.04	63.2%	
Gender						
Both sexes	5	1.84(0.49, 3.19)	**0.007**	0.22	28.9%	0.08
Male	2	0.65(−0.37, 1.68)	0.21	0.87	0.0%	
Female	1	−1.66(−4.59, 1.27)	0.26	–	–	
Baseline BMI (Kg/m^2^)						
Normal	1	0.80 (−1.31, 2.91)	0.45	–	–	0.22
Overweight	3	2.02 (0.36, 3.67)	**0.01**	0.10	56.2%	
Obesity	4	0.28 (−0.77, 1.33)	0.60	0.55	0.0%	
Subgroup analyses of raspberry consumption on AST (U/L)						
Overall effect	4	−2.01(−4.67, 0.64)	0.13	**0.004**	77.8%	
Country						
Korea	2	−2.84(−10.38, 4.70)	0.46	**0.03**	77.5%	0.78
None-Korea	2	−1.70(−4.84, 1.42)	0.28	**0.003**	88.8%	
Duration (weeks)						
≤8	1	−3.30(−4.75, −1.84)	**<0.001**	–	–	0.37
>8	3	−1.48(−5.24, 2.26)	0.43	0.05	66.1%	
Raspberry type						
Black raspberry	2	−2.84(−10.38, 4.70)	0.46	**0.03**	77.5%	0.78
None-black raspberry	2	−1.70(−4.84, 1.42)	0.28	**0.003**	88.8%	
Health status						
Healthy	1	−0.10(−1.61, 1.41)	0.89	–	–	0.01
Prediabetes	2	−2.84(−10.38, 4.70)	0.46	**0.03**	77.5%	
Type 2 diabetes melitus	1	−3.30(−4.75, −1.84)	**<0.001**	–	–	
Age						
≤35	1	−0.10(−1.61, 1.41)	0.89	–	–	0.11
>35	3	−2.99(−6.25, 0.27)	0.07	0.09	56.8%	
Gender						
Both sexes	3	−2.99(−6.25, 0.27)	0.07	0.09	56.8%	0.11
Female	1	−0.10(−1.61, 1.41)	0.89	–	–	
Baseline BMI (Kg/m^2^)						
Overweight	2	−2.84(−10.38, 4.70)	0.46	**0.03**	77.5%	0.78
Obesity	2	−1.70(−4.84, 1.42)	0.28	**0.003**	88.8%	
Baseline AST						
≤30	3	−1.48(−5.24, 2.26)	0.43	0.05	66.1%	0.37
>30	1	−3.30(−4.75, −1.84)	**<0.001**	–	–	
Subgroup analyses of raspberry consumption on ALT (U/L)						
Overall effect	4	−0.85(−2.10, 0.40)	0.18	0.30	16.9%	
Country						
Korea	2	−2.13(−5.17, 0.89)	0.16	0.28	13.5%	0.35
None-Korea	2	−0.55(−1.96, 0.84)	0.43	0.22	30.8%	
Duration (weeks)						
≤8	1	−1.20(−2.72, 0.32)	0.12	–	–	0.78
>8	3	−0.82(−3.03, 1.38)	0.46	0.20	36.2%	
Raspberry type						
Black raspberry	2	−2.13(−5.17, 0.89)	0.16	0.28	13.5%	0.35
None-black raspberry	2	−0.55(−1.96, 0.84)	0.43	0.22	30.8%	
Health status						
Healthy	1	0.24(−1.54, 2.02)	0.79	–	–	0.31
Prediabetes	2	−2.13(−5.17, 0.89)	0.16	0.28	13.5%	
Type 2 diabetes melitus	1	−1.20(−2.72, 0.32)	0.12	–	–	
Age						
≤35	1	0.24(−1.54, 2.02)	0.79	–	–	0.14
>35	3	−1.41(−2.75, −0.07)	**0.03**	0.47	0.0%	
Gender						
Both sexes	3	−1.41(−2.75, −0.07)	**0.03**	0.47	0.0%	0.14
Female	1	0.24(−1.54, 2.02)	0.79	–	–	
Baseline BMI (Kg/m^2^)						
Overweight	2	−2.13(−5.17, 0.89)	0.16	0.28	13.5%	0.35
Obesity	2	−0.55(−1.96, 0.84)	0.43	0.22	30.8%	
Baseline ALT (U/L)						
≤30	3	−0.82(−3.03, 1.38)	0.46	0.20	36.2%	0.78
>30	1	−1.20(−2.72, 0.32)	0.12	-	-	

CI, confidence interval; WMD, weighted mean differences; METS, metabolic syndrome; BMI, Body Mass Index; parallel, R, PC, DB, parallel, randomized placebo controlled double blind; parallel, R, PC, SB, parallel, randomized placebo controlled single blind; Cross over, R, PC, DB, cross over, randomized placebo controlled double blind; WC, waist circumference; AST, aspartate transaminase; ALT, alanine transaminase. BMI classification: in Korean population: 18.5–22.9 Kg/m^2^: normal; 23–24.9 Kg/m^2^: overweight; 25 Kg/m^2^≤: obesity. In non-Korean population: 18.5–24.9 Kg/m^2^: normal; 25–29.9 Kg/m^2^: overweight; 30 Kg/m^2^≤: obesity. Bolds indicates statistically significant.

#### 3.4.2 Effect of raspberry consumption on BMI

The combination of seven effect sizes with 256 participants demonstrated that raspberry consumption had no significant effect on BMI compared to control groups [WMD, −0.18 Kg/m^2^; 95%CI, (−0.41 to 0.04); *p* = 0.11; 256 participants] (Figure 3B). Also, no significant heterogeneity was detected among the included studies (*I*^2^ = 8.6%, *p* = 0.36). Subgroup analysis showed that raspberry consumption did not significantly change BMI in any of the subgroups (Table 2).

#### 3.4.3 Effect of raspberry consumption on WC

Performing a meta-analysis on eight effect sizes with 295 participants showed no significant effect of raspberry consumption on WC compared to control groups [WMD, 1.06 cm; 95%CI, (−0.005 to 2.13); *p* = 0.05; 295 participants] (Figure 3C). In addition, there was no significant heterogeneity among the included studies (*I*^2^ = 44.9%, *p* = 0.08). Subgroup analysis mentioned a significantly increasing effect of raspberry intake in studies conducted in Korea, on both sexes, or those participants that consumed black raspberry, or in individuals with prediabetes, aged >35 years, or overweight (Table 2).

#### 3.4.4 Effect of raspberry consumption on AST

By combining four effect sizes with 99 participants, it was shown that raspberry consumption did not have a significant effect on AST compared to the control groups [WMD, −2.01 U/L; 95%CI, (−4.67 to 0.64); *p* = 0.13; 99 participants] (Figure 3D). However, significant heterogeneity was identified between the included studies (*I*^2^ = 77.8%, *p* = 0.004). Subgroup analysis noted that raspberry consumption led to a significant reduction in AST levels in studies with ≤8 weeks duration, conducted on individuals with type 2 diabetes mellitus, or in individuals with more than 30 U/L baseline AST levels (Table 2).

#### 3.4.5 Effect of raspberry consumption on ALT

Pooling four effect sizes with 99 participants, revealed that raspberry consumption did not have a significant effect on ALT compared to the control groups [WMD, −0.85 U/L; 95%CI, (−2.10 to 0.40); *p* = 0.18; 99 participants] (Figure 3E). Furthermore, no significant heterogeneity was observed between the included studies (*I*^2^ = 16.9%, *p* = 0.30). Subgroup analysis showed that consumption of raspberry in individuals aged >35 years or in studies on both sexes significantly decreased the ALT levels (Table 2).

### 3.5 Publication bias

**Figure 4:**
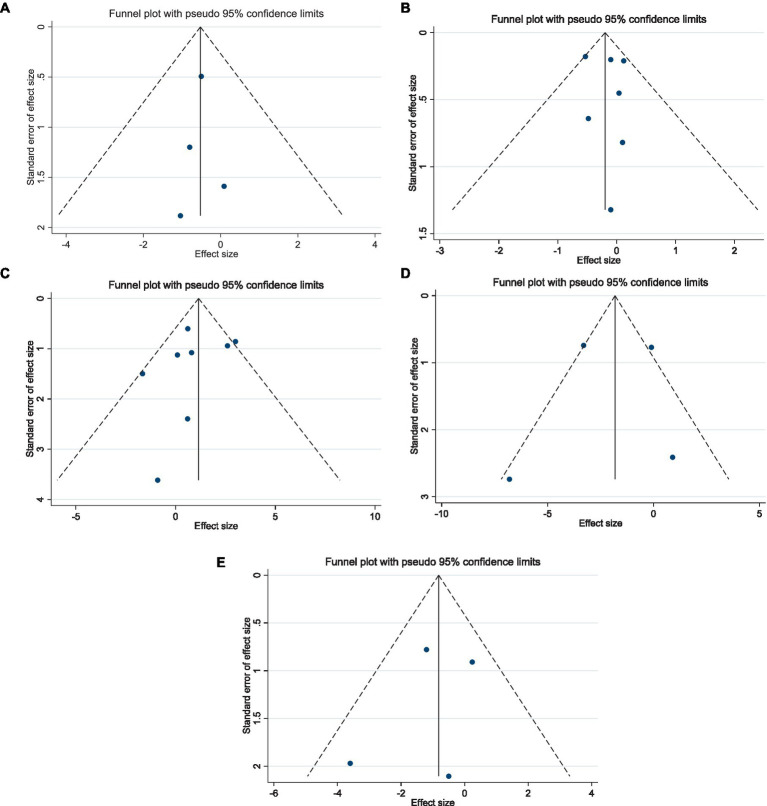
Funnel plots for the effect of raspberry intake on **(A)** weight (Kg); **(B)** body mass index (Kg/m^2^); **(C)** waist circumference (cm); **(D)** aspartate transaminase (U/L), and **(E)** alanine transaminase (U/L).

Egger’s regression test and visual interpretation of funnel plots demonstrated that there was no significant publication bias in the included studies investigating the effect of raspberry consumption on Weight (*P*_Egger_: 0.86), BMI (*P*_Egger_: 0.71), WC (*P*_Egger_: 0.51), AST (*P*_Egger_: 0.86), and ALT (*P*_Egger_: 0.60) (Figures 4A–E).

### 3.6 Sensitivity analysis

The sensitivity analysis, conducted to determine the influence of each of the included effect sizes on the overall effect size, demonstrated the pooled effect size of raspberry consumption on BMI after omitting An et al. (31) (WMD: −0.30 kg/m^2^, 95%CI: −0.54, −0.06) and for WC after excluding Khazaal et al. (29) (WMD: 1.36 cm, 95%CI: 0.37, 2.34) changed significantly. Furthermore, excluding the study by Mosah et al. (30) (WMD: −1.41 U/L, 95%CI: −2.75, −0.07) led to a significant change in the overall size effect of raspberry consumption on ALT levels (Supplementary Figures 1A–E).

### 3.7 GRADE analysis

The certainty quality of the evidence included in this review was evaluated by following the GRADE protocol (26). The quality of evidence investigating the effect of raspberry intake on weight, BMI, WC, and ALT was downgraded to low due to serious risk of bias and imprecision. Furthermore, the quality of evidence for AST was downgraded to very low due to serious Imprecision, serious risk of bias, and very serious inconsistency. The GRADE profile for anthropometric indices and liver function tests is presented in Table 3.

**Table 3:** GRADE profile of raspberry consumption for lipid profile and blood pressure.

Outcomes	Risk of bias	Inconsistency	Indirectness	Imprecision	Publication Bias	Quality of evidence
Weight (Kg)	Serious limitations	No serious limitations	No serious limitations	Serious limitations^b^	No serious limitations	⊕⊕◯◯ Low
BMI (Kg/m^2^)	Serious limitations	No serious limitations	No serious limitations	Serious limitations^b^	No serious limitations	⊕⊕◯◯ Low
WC (cm)	Serious limitations	No serious limitations	No serious limitations	Serious limitations^b^	No serious limitations	⊕⊕◯◯ Low
AST (U/L)	Serious limitations	Very serious limitations^a^	No serious limitations	Serious limitations^b^	No serious limitations	⊕◯◯◯ Very low
ALT (U/L)	Serious limitations	No serious limitations	No serious limitations	Serious limitations^b^	No serious limitations	⊕⊕◯◯ Low

CI, confidence interval; WMD, weighted mean differences; BMI, Body Mass Index; WC, Waist circumference; AST, aspartate transaminase; ALT, alanine transaminase. ^a^There is high heterogeneity (*I*^2^ > 75%). ^b^There is no evidence of significant effects of raspberry intake.

## 4 Discussion

The current systematic review and meta-analysis were performed to summarize the results of nine randomized controlled studies (10 arms) with 355 participants regarding the effects of raspberry on weight, BMI, WC, AST, and ALT levels. Our results demonstrated no significant changes in any of the outcomes following raspberry consumption. Moreover, subgroup analysis showed that duration of intervention, age, type of raspberry, and individual characteristics could change the results of raspberry intake on WC and liver enzymes. For example, raspberry had promising effects on AST levels in individuals with type 2 diabetes Mellitus, baseline AST levels higher than 30 U/L, or in ≤8 weeks durations. Also, raspberry reduced ALT levels in individuals older than 35 years old. To our knowledge, this is the first meta-analysis that studied the effects of raspberry on liver enzymes, weight, WC, and BMI with comprehensive subgroup analysis. In this regard, a previous meta-analysis (with 22 RCTs) was conducted on all berries consumption and did not consider their specific type, such as raspberry (35). Our findings were inconsistent with this review which reported a significant decrease in BMI following berry consumption (35).

Raspberry is a popular rich source of several bioactive compounds such as vitamin C, niacin, phenolic compounds, and organic acids like anthocyanins, resveratrol, ellagic acid, gallic acid, caffeic acid, epicatechin, quercetin, lambertianin, and kaempferol (36, 37). However, previous meta-analyses of the bioactive compounds of berries were consistent with our results (38–40). A meta-analysis of 15 trials with 714 participants mentioned no significant effects of resveratrol on AST and ALT levels which is consistent with our results (39). Also, consistent with our findings regarding BMI, a meta-analysis of 21 studies showed no significant effects of anthocyanin-rich berries on metabolic syndrome risk factors such as BMI (38). Another meta-analysis of 44 RCTs and 15 prospective cohort studies exploring the effects of anthocyanin-rich berries on cardiovascular risk reported no substantial improvements in BMI (40).

Despite raspberry’s antioxidant and anti-inflammatory compounds, our findings demonstrate no significant improvement in body weight, BMI, WC, AST, and ALT indices. Seasonal changes in the dietary habits of individuals may affect the intake of phenolic compounds and organic acids in both placebo and intervention groups of considered studies, which can influence the results. Also, dietary intake of other types of berries may cause synergistic or weakening effects on findings. As a result, we cannot consider all the findings to raspberry alone. Moreover, some flavors and colorings used to prepare placebo are derived from fruits commonly berries. Thus, the placebo and intervention group may not exhibit the real effects of raspberry. However, controlling all these parameters is difficult in dietary interventions.

The present meta-analysis had several strengths. Raspberry is a rich source of bioactive compounds with health improvement effects, however, its exact effect on weight, WC, and liver enzymes had not been studied. This meta-analysis summarized the results of all clinical trials in this regard for the first time. Moreover, a comprehensive risk of bias assessment of studies was conducted using the updated guideline proposed by the Cochrane Collaboration. Also, a general search for studies using MeSH and Non-MeSH terms, sensitivity analysis to assess the stability of results, and a comprehensive subgroup analysis based on the possible source of heterogeneity. However, this study had limitations. The included studies had different target populations, such as studies had different target populations, such as individuals with risk of metabolic syndrome, metabolic syndrome, type 2 diabetes, prediabetes, andropause symptoms, or healthy people, which may have confounding effects on our findings. Moreover, the general risk of bias for the majority of included trials was identified as high. In addition, the quality of the evidence for body weight, BMI, WC, and ALT was downgraded to low due to serious imprecision and risk of bias, and the quality of the evidence for AST was downgraded to very low due to serious imprecision, risk of bias, and very serious inconsistency. Another limitation of this review is that the power of studies was not reported in the majority of the included trials, which need to interpret their results with caution.

## 5 Conclusion

In conclusion, this systematic review and meta-analysis revealed the non-significant effect of raspberry intake on anthropometric indices such as weight, BMI, WC, and liver function tests such as AST and ALT levels. However, it is suggested to conduct more high-quality RCTs with larger sample sizes to investigate the impact of each type of raspberry on different populations before drawing a definite conclusion. Also, it is recommended to evaluate changes in body composition after raspberry consumption in the future.

## Data availability statement

The original contributions presented in the study are included in the article/Supplementary material, further inquiries can be directed to the corresponding author.

## Author contributions

MShJ: Conceptualization, Formal analysis, Investigation, Methodology, Project administration, Visualization, Writing – original draft, Writing – review & editing. HB: Data curation, Investigation, Methodology, Software, Writing – original draft. PR: Data curation, Formal analysis, Methodology, Project administration, Visualization, Writing – original draft. NP: Investigation, Methodology, Project administration, Supervision, Writing – review & editing.
