# Supplementary material for: The effects of raspberry consumption on anthropometric indices and liver function tests in adults: a GRADE-assessed systematic review and meta-analysis
Source: Front Nutr. 2024 Aug 5;11:1419417. doi: 10.3389/fnut.2024.1419417 (PMC11332610; doi:10.3389/fnut.2024.1419417)

**Supplementary Table 1.** Details of the search strategy in each database

| **Database** | **TERMS** |
| --- | --- |
| PubMed | **(**(((("Raspberry"[Title/Abstract]) OR ("Rubus Occidentalis"[Title/Abstract])) OR ("Rubus idaeus"[Title/Abstract])) OR ("Rubus coreanus"[Title/Abstract])) AND ((((((((((("Liver enzymes"[Title/Abstract]) OR ("Hepatic enzymes"[Title/Abstract])) OR ("Aspartate aminotransferase"[Title/Abstract])) OR ("AST"[Title/Abstract])) OR ("Alanine aminotransferase"[Title/Abstract])) OR ("ALT"[Title/Abstract])) OR ("Alkaline phosphatase"[Title/Abstract])) OR ("ALP"[Title/Abstract])) OR ("Gamma-glutamyl transpeptidase"[Title/Abstract])) OR ("GGT"[Title/Abstract])) OR ((((((((((((("Body Weight"[Title/Abstract]) OR ("Body Mass Index"[Title/Abstract])) OR ("BMI"[Title/Abstract])) OR ("Weight Loss"[Title/Abstract])) OR ("Waist Circumference"[Title/Abstract])) OR ("WC"[Title/Abstract])) OR ("Hip Circumference"[Title/Abstract])) OR ("HC"[Title/Abstract])) OR ("Quetelet Index"[Title/Abstract])) OR ("Weight Reduction"[Title/Abstract])) OR ("Overweight"[Title/Abstract])) OR ("Obesity"[Title/Abstract])))) AND ((((((((((((((randomized[Title/Abstract]) OR (placebo[Title/Abstract])) OR (clinical trials[Title/Abstract])) OR (randomly[Title/Abstract])) OR (trial[Title/Abstract])) OR (randomized controlled trial[Title/Abstract])) OR (RCT[Title/Abstract])) OR (("Clinical Trials as Topic"[Mesh]) OR ( "Clinical Trial" [Publication Type] OR "Controlled Clinical Trial" [Publication Type] ))))))))) |
| SCOPUS | ( ( TITLE-ABS-KEY ( "Raspberry" ) OR TITLE-ABS-KEY ( "Rubus Occidentalis" ) OR TITLE-ABS-KEY ( "Rubus idaeus" ) OR TITLE-ABS-KEY ( "Rubus coreanus" ) ) ) AND ( ( ( TITLE-ABS-KEY ( "Liver enzymes" ) OR TITLE-ABS-KEY ( "Hepatic enzymes" ) OR TITLE-ABS-KEY ( "Aspartate aminotransferase" ) OR TITLE-ABS-KEY ( "AST" ) OR TITLE-ABS-KEY ( "Alanine aminotransferase" ) OR TITLE-ABS-KEY ( "ALT" ) OR TITLE-ABS-KEY ( "Alkaline phosphatase" ) OR TITLE-ABS-KEY ( "ALP" ) OR TITLE-ABS-KEY ( "Gamma-glutamyl transpeptidase" ) OR TITLE-ABS-KEY ( "GGT" ) ) ) OR ( ( TITLE-ABS-KEY ( "Body Weight" ) OR TITLE-ABS-KEY ( "Body Mass Index" ) OR TITLE-ABS-KEY ( "BMI" ) OR TITLE-ABS-KEY ( "Weight Loss" ) OR TITLE-ABS-KEY ( "Waist Circumference" ) OR TITLE-ABS-KEY ( "WC" ) OR TITLE-ABS-KEY ( "Hip Circumference" ) OR TITLE-ABS-KEY ( "HC" ) OR TITLE-ABS-KEY ( "Quetelet Index" ) OR TITLE-ABS-KEY ( "Weight Reduction" ) OR TITLE-ABS-KEY ( "Overweight" ) OR TITLE-ABS-KEY ( "Obesity" ) ) ) ) AND ( ( ( ( ( ( TITLE-ABS-KEY ( randomized ) OR TITLE-ABS-KEY ( placebo ) OR TITLE-ABS-KEY ( clinical AND trials ) OR TITLE-ABS-KEY ( randomly ) OR TITLE-ABS-KEY ( trial ) OR TITLE-ABS-KEY ( randomized AND controlled AND trial ) OR TITLE-ABS-KEY ( rct ) ) ) ) ) ) ) |
| Web of Science | **Query #1:**  (((TS=("Raspberry")) OR TS=("Rubus Occidentalis")) OR TS=("Rubus idaeus")) OR TS=("Rubus coreanus")  **Query #2:**  (((((((((TS=("Liver enzymes" )) OR TS=("Hepatic enzymes" )) OR TS=("Aspartate aminotransferase" )) OR TS=("AST")) OR TS=("Alanine aminotransferase" )) OR TS=("ALT")) OR TS=("Alkaline phosphatase")) OR TS=("ALP")) OR TS=("Gamma-glutamyl transpeptidase")) OR TS=(GGT)  **Query #3**  (((((((((((TS=("Body Weight")) OR TS=("Body Mass Index")) OR TS=("BMI")) OR TS=("Weight Loss")) OR TS=("Waist Circumference")) OR TS=("WC")) OR TS=("Hip Circumference")) OR TS=("HC")) OR TS=("Quetelet Index")) OR TS=("Weight Reduction")) OR TS=("Overweight")) OR TS=("Obesity")  **Query #4:**  Query #2 OR Query #3  **Query #5:**  ((((((TS=(randomized)) OR TS=("placebo")) OR TS=("clinical trials")) OR TS=("randomly")) OR TS=("trial")) OR TS=("randomized controlled trial")) OR TS=("RCT")  **Final:**  **Query #1 AND Query #4 AND Query #5** |

Supplementary Figure 1. Influence of omitting each effect size on the overall effect sizes of the impact of raspberry on A) Weight (Kg); B) Body mass index (Kg/m^2^); C) Waist circumference (cm); D) Aspartate transaminase (U/L), and E) Alanine transaminase (U/L).

**A)**

B)

C)

D)

E)

Supplementary Figure 2. Summary plot of risk of bias assessment


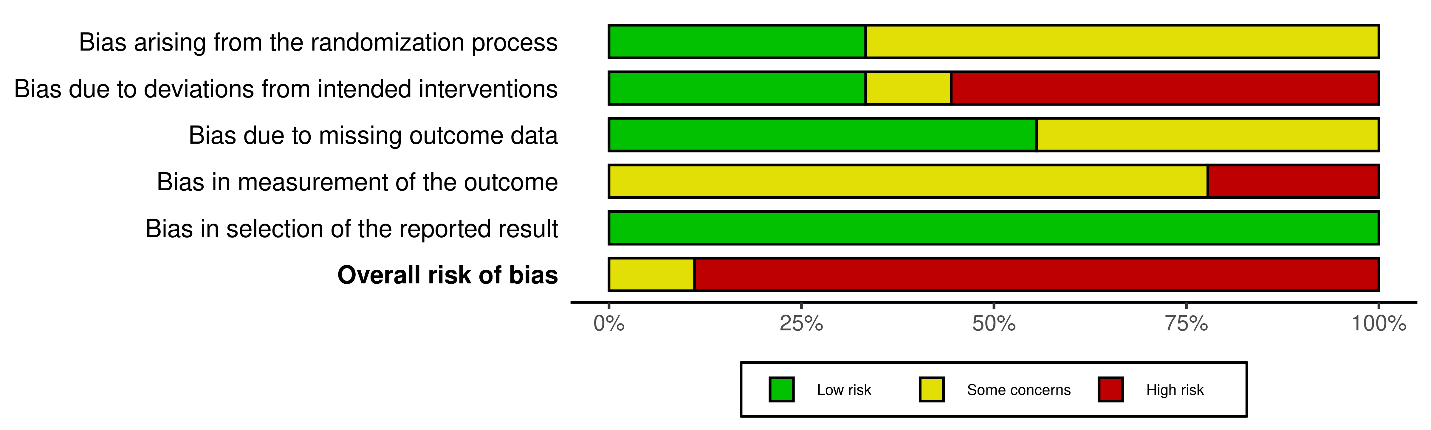

Supplement: Supplementary file 1 [file Data_Sheet_1.docx]
